# Effects of Microplastics and Cadmium on the *Leptinotarsa decemlineata* (Coleoptera: Chrysomelidae): An Evaluation Using a Two-Sex Life Table

**DOI:** 10.3390/insects17060638

**Published:** 2026-06-17

**Authors:** Boling Liu, Yunhui Liu, Yi Zhang, Bingyu He, Yulin Gao, Chao Li

**Affiliations:** 1 Key Laboratory of Prevention and Control of Invasive Alien Species in Agriculture & Forestry of the North-Western Desert Oasis, Ministry of Agriculture and Rural Affairs, College of Agronomy, Xinjiang Agricultural University, Urumqi 830052, China; liuboling2001@163.com (B.L.); 15199103540@163.com (Y.L.); 15071785641@vip.163.com (Y.Z.); 18040907242@163.com (B.H.); 2 State Key Laboratory for Biology of Plant Diseases and Insect Pests, Institute of Plant Protection, Chinese Academy of Agricultural Sciences, Beijing 100193, China; gaoyulin@caas.cn; 3 Institute of Bast Fiber Crops & Center of Southern Economic Crops, Chinese Academy of Agricultural Sciences, Changsha 410205, China

**Keywords:** microplastics, heavy metals, life tables, *Leptinotarsa decemlineata*

## Abstract

This study utilized the age-stage, two-sex life table method to evaluate the toxic effects of polyethylene microplastics (PE, 300 mg/kg) and cadmium (Cd, 30 mg/kg), both individually and combined, on *Leptinotarsa decemlineata*. Compared to controls, all treatments significantly prolonged larval development and reduced survival, lifespan, and fecundity. The combined exposure (PE + Cd) exerted the strongest inhibition: the total pre-adult developmental duration (TPOP) increased by 18.8% (38.00 days), while the intrinsic growth rate (r) dropped by 59.0% to 0.0273 d^−1^. Additionally, the net reproduction rate ($R_{0}$) and fecundity fell to their lowest levels (5.08 and 19.06, respectively), significantly lower than in single-treatment groups. Age-stage life expectancy analysis confirmed severe survival pressure in the combined group, evidenced by a 30% reduction in first-instar survival and a 14-day shortened adult lifespan. These findings demonstrate the synergistic toxicity of PE and Cd co-contamination, providing critical data for ecological risk assessment in the “soil–plant–herbivore” system and integrated pest management strategies.

## Simple Summary

Soil pollution caused by microplastics and heavy metals has become a global agricultural threat, but their combined effects on key agricultural pests remain unclear. This study used a two-sex life table to evaluate the impacts of polyethylene microplastics (PE) and cadmium (Cd), alone and in combination, on the growth, development, and population dynamics of the *Leptinotarsa decemlineata*. The results showed that single PE or Cd stress significantly prolonged larval development, reduced survival and fecundity, and inhibited population growth. The PE + Cd combined treatment had a combined inhibitory effect, causing the strongest decline in key population parameters. These findings clarify the combined toxic risk of microplastics and cadmium to insect pests, provide a scientific basis for ecological risk assessment of soil pollutants, and support the optimization of integrated pest management strategies for potatoes.

## 1. Introduction

*Leptinotarsa decemlineata* (Say) (Coleoptera: Chrysomelidae, Chrysomelinae) is a major destructive phytophagous pest of solanaceous crops. It is a devastating polyphagous pest in potato-growing regions worldwide. Larvae and adults feed primarily on potato leaves, thereby damaging the photosynthetic organs, which results in yield losses ranging from 30% to 100% under severe infestations. Such yield loss, for instance, poses a direct threat to the industrial security of major potato-producing areas in Northwestern China [1,2,3]. Elucidating the developmental rhythm and population growth potential of the *Leptinotarsa decemlineata* constitutes a fundamental prerequisite for devising precise control strategies.

The rapid transformation of agricultural production has brought challenges such as soil pollution from multiple sources. For example, polyethylene microplastics and heavy metal cadmium have become key pollutants threatening the health of farmland soil ecosystems due to their environmental persistence, bioaccumulation potential, and cross-trophic level toxicity [4,5]. Microplastics originate from a wide range of sources, including the aging and degradation of agricultural mulch film, fragmentation of plastic waste, introduction via organic fertilizers, and atmospheric dry and wet deposition. Characterized by their small particle size and large specific surface area, they readily adsorb to soil particles or are absorbed by plant roots, thereby entering the food chain through the soil–plant system [6,7,8]. They significantly inhibit potato germination and growth, reduce yield, and, through bottom-up effects along the food chain, decrease the survival rate and fecundity of *Leptinotarsa decemlineata* adults while prolonging larval development duration. Simultaneously, through trophic transfer, they significantly increase the mortality of both nymph and adult stages of the natural predator *Arma chinensis* (Hemiptera: Pentatomidae). Although they shorten nymphal developmental time and increase adult body weight, they markedly extend predation time and reduce searching and paralyzing efficiency, ultimately weakening the biocontrol capacity of *A. chinensis* against the *Leptinotarsa decemlineata* [9,10].

Cadmium, a highly toxic heavy metal, is continuously introduced into agricultural soils through industrial emissions, excessive application of chemical fertilizers, and wastewater irrigation, characterized by high mobility, prolonged retention periods, and a strong propensity for crop uptake and accumulation [11,12,13,14]. Cadmium stress not only inhibits plant photosynthesis and nutrient uptake but can also be transferred along the food chain and disrupt the antioxidant systems and energy metabolism of insects, significantly reducing their survival and reproductive capacity. Studies have confirmed that cadmium stress significantly prolongs the duration of the first-instar larval stage, pupal stage, and pre-oviposition period of the *Leptinotarsa decemlineata*, shortens adult longevity, reduces fecundity, decreases survival rates across all life stages, and increases the deformity rate of newly emerged adults. Population life table parameters reveal that increase in cadmium concentration is associated with significant decline in the intrinsic rate of increase, finite rate of increase, net reproductive rate, and gross reproductive rate of the beetle, while the mean generation time shows no significant change, indicating that cadmium exerts a pronounced inhibitory effect on the development, survival, reproduction, and population growth of the *Leptinotarsa decemlineata* through plant-mediated pathways [15,16,17].

Multiple pollutants affect agricultural environments. Therefore, assessing the effects of single pollutants is insufficient to reflect actual ecological risks. Current research has largely focused on the individual toxic effects of microplastics or cadmium on plants and soil organisms, whereas systematic studies on their combined effects on the growth, development, life-history traits, and population dynamics of the *Leptinotarsa decemlineata* remain scarce [18]. Microplastics and cadmium interact through surface adsorption, complexation, and other mechanisms, altering their environmental behavior and bioavailability, thereby producing combined, antagonistic, or additive effects [19]. Their coexistence in agricultural soils is becoming increasingly common, and elucidating their sublethal effects on key insect pests can provide a basis for predicting pest outbreak trends, optimizing pesticide application, and guiding ecological remediation [20,21]. This study aims to fill the research gap regarding the impacts of combined microplastic and cadmium pollution on *Leptinotarsa decemlineata* and to provide scientific support for optimizing integrated management strategies for *Leptinotarsa decemlineata* exposed to multiple pollutants in the major potato-producing regions of Northwestern China.

The response of insect populations to environmental pollutants is a key topic at the intersection of ecotoxicology and integrated pest management (IPM). Elucidating how pollutants affect insect life-history traits and population parameters helps improve ecological risk assessment models. If exposure to pollution reduces the survival and reproductive capacity of *Leptinotarsa decemlineata*, this suggests that soil pollution may alter pest population dynamics to some extent; conversely, if pollution enhances their tolerance, compensatory feeding capacity, or risk of pesticide resistance, this indicates that traditional pest control strategies may prove ineffective or produce unintended consequences in polluted farmland. Therefore, in agricultural practice, it is necessary not only to focus on the direct impact of pollution on crop yield and quality but also to incorporate it into pest monitoring, early warning, and green pest control systems. This requires a comprehensive consideration of the interrelationships among soil remediation, reduction in plastic use in farmland, control of heavy metal inputs, and ecological responses of pests, thereby providing more forward-looking scientific evidence for farmland ecological safety and sustainable agricultural management under conditions of pollution.

## 2. Materials and Methods

### 2.1. Potato Cultivation

To ensure that the experiments can effectively reveal the ecological effects of pollutants, relevant studies have reported that the average amount of plastic film residue in Chinese farmland is 103 kg/hm^2^, while the average abundance of microplastics reaches 4537 particles/kg dry soil [22]. Our team has previously conducted separate studies on the effects of cadmium and polyethylene microplastics on the *Leptinotarsa decemlineata* and its natural enemies. Zhang [10] found that potato growth showed a concentration-dependent response to polyethylene microplastics (5 μm), and that exposure to 300 mg/kg significantly reduced the activity and survival rate of the *Leptinotarsa decemlineata*. He reported that cadmium exposure at 30 mg/kg significantly affected the activity and survival of the *Leptinotarsa decemlineata* [12].

“Wotu No. 5” potatoes were grown in 30 cm-diameter round pots, and leaves were collected under different treatment conditions to feed the *Leptinotarsa decemlineata*. The experiment consisted of four soil treatments, each with 15 replicates (pots) to assess the individual and combined effects of cadmium (Cd) and microplastics (MPs) on plant and insect:

Treatment A (CK): No additives (control).

Treatment B (Cd): Addition of cadmium chloride at a concentration of 30 mg/kg.

Treatment C (PE): Addition of 5 μm polyethylene microplastics at a concentration of 300 mg/kg.

Treatment D (Cd + PE): Combined application of 30 mg/kg Cd and 300 mg/kg 5 μm polyethylene microplastics.

The growth substrate was sterilized prior to treatment. Cadmium was applied by spraying with a cadmium chloride (Tongluoma Technology Co., Ltd., Beijing, China) solution, followed by thorough mixing, while microplastics were dry-blended into the soil. The treated soil was turned over daily and allowed to equilibrate for two weeks.

Each pot was filled with 3 kg of experimental soil, which was sandy in texture, had a moisture content of 20 ± 5%, a pH of 7.8, and an organic matter content of 11 g/kg. The soil was purchased from the Mingzhu Flower Market in Urumqi, Xinjiang. Uniform seed potatoes were selected, with one plant planted per pot. All pots were placed outdoors at Xinjiang Agricultural University in Urumqi, Xinjiang, and managed under standard conditions, with regular watering to maintain soil moisture. No fertilizers or pesticides were applied throughout the entire growing season.

### 2.2. Insect Feeding

The founding population of *Leptinotarsa decemlineata* used in this experiment was collected in May 2025 from a potato field in Liuchengzi West Village, Fukang City, Changji Prefecture, Xinjiang (87.92° E, 44.18° N). The captured adults were kept in an intelligent artificial climate chamber and reared continuously for generations with fresh, uncontaminated potato leaves. The rearing conditions were kept constant at a temperature of 28 ± 1 °C, relative humidity of 50% ± 5%, and a photoperiod of L:D = 16:8. Potato leaves were replaced daily to ensure sufficient fresh food. The second-generation population was used for the experiment. Egg masses deposited by second-generation adults were monitored, and newly hatched first-instar larvae of the same age were then selected for subsequent experiments.

Larvae were fed with leaves from the treated plants. Each treatment had four replicates, and each replicate consisted of 30 randomly selected newly hatched first-instar larvae. Larvae were group-reared in Petri dishes and provided with fresh potato leaves daily. To maintain leaf turgor, the petioles were wrapped with moistened absorbent cotton. A layer of moist sterile sand (approximately 5 cm deep) was provided in each cup to serve as a suitable pupation medium. The emergence time of each adult was recorded. Newly emerged adults of the same age were paired in male–female pairs at a 1:1 ratio. Rearing conditions were maintained at 28 ± 1 °C, 50% ± 5% relative humidity, and a photoperiod of L:D = 16:8.

When possible, pairing priority individuals originated from the same replicate group. If a replicate group lacked a male, a substitute was selected from another replicate group within the same treatment that had a comparable developmental timeline. The survival and fecundity (number of egg masses and total eggs) of all paired adults were monitored daily [23]. The recording of mortality differed for the different sexes. In the event of male mortality, replacements were made from the same treatment, and their survival days were no longer recorded. Similarly, if females died, their egg-laying activity and survival days were no longer tracked [12].

### 2.3. Statistical Analysis

Data were managed in Microsoft Excel and statistically analyzed using IBM SPSS Statistics 26.0 with Duncan’s test for group comparisons. The life table parameters for *Leptinotarsa decemlineata*, developmental time, survival, and population growth indices (innate growth rate *r*, finite growth rate *λ*, net reproductive rate *R*_0_, mean generation time *T*, and gross reproduction rate GRR) were analyzed using TWOSEX-MS Chart software (1 December 2024) [24,25]. Treatment differences for these parameters were evaluated using paired-wise tests with 100,000 bootstrap replications at a 5% significance level. GraphPad Prism10.3.0 software was used for plotting.

The primary formulas are as follows:

Age-Specific Survivability (*l_x_*): $l_{x}=\sum_{j=1}^{m}\;S_{xj}$

Age-Stage-Specific Fecundity (*m_x_*): $m_{x}=\frac{\sum_{j=1}^{m}\;S_{xj}f_{xj}}{\sum_{j=1}^{m}\;S_{xj}}$

Age-Stage Life Expectancy (*e_xj_*): $e_{xj}=\sum_{i=x}^{\infty }\;\sum_{y=j}^{m}\;{S^{\prime }}_{iy}$

Intrinsic Rate of Increase (*r*): $\sum_{x=0}^{\infty }\;e^{-r(x+1)}l_{x}m_{x}=1$

Finite Rate of Increase (*λ*): $\lambda =e^{r}$

Net Reproductive Rate (*R*_0_): $R_{0}=\sum_{x=0}^{\infty }\;l_{x}m_{x}$

Mean Generation Time (*T*): $T=\frac{lnR_{0}}{r}$

Gross reproduction rate (*GRR*): $GRR=\sum m_{x}$

## 3. Results

### 3.1. Effects of Different Treatments on the Developmental Duration of the Leptinotarsa decemlineata

Single and combined treatments of PE and Cd significantly affected the developmental duration of *Leptinotarsa decemlineata* across all larval stages (Table 1). Compared to the control (CK), all treatments prolonged larval development.

Specifically, the L1 stage lasted 1.04 ± 0.04 d in CK, but extended to 2.96 ± 0.22 d under PE treatment (184% increase, *p* < 0.001), 2.67 ± 0.09 d under Cd (157% increase), and 2.74 ± 0.08 d under PE + Cd (163% increase). In the L2 stage, PE caused the most pronounced delay (4.78 ± 0.09 d) compared to CK (2.31 ± 0.09 d), representing a 107% prolongation (*p* < 0.001). The combined treatment (PE + Cd) also significantly extended L2 duration to 3.5 ± 0.11 d (51% increase, *p* < 0.01). For L3 and L4, similar patterns were observed, with PE treatment showing the strongest effects. The pupal duration increased from 9.67 ± 0.26 d (CK) to 11.55 ± 0.18 d under PE (19% increase, *p* < 0.001), while Cd and PE + Cd also caused significant prolongations (10.67 ± 0.28 d and 10.5 ± 0.32 d, respectively). Adult longevity did not differ significantly among groups (*p* > 0.05). The total pre-oviposition period (TPOP) increased from 32.0 ± 0.41 d (CK) to 38.92 ± 0.60 d under PE (22% increase, *p* < 0.001) and to 38.0 ± 0.80 d under PE + Cd (19% increase, *p* < 0.05).

### 3.2. Effects of Different Treatments on Life Table Parameters of Leptinotarsa decemlineata Populations

All treatments significantly suppressed population growth and reproductive potential compared to CK (Table 2). The intrinsic rate of increase (r) in CK was 0.066 ± 0.0039 d^−1^. PE alone reduced r by 38% to 0.041 ± 0.0042 d^−1^ (*p* < 0.001), and Cd alone reduced it by 27% to 0.049 ± 0.005 d^−1^ (*p* < 0.01). The combined PE + Cd treatment caused the strongest inhibition, reducing r by 59% to 0.027 ± 0.0058 d^−1^ (*p* < 0.001). Similarly, the finite rate of increase (λ) decreased from 1.07 ± 0.0042 (CK) to 1.028 ± 0.0059 under PE + Cd (*p* < 0.001).

The net reproductive rate (R_0_) declined from 20.12 ± 3.19 (CK) to 9.16 ± 1.91 under PE (54% reduction), 9.79 ± 2.09 under Cd (51% reduction), and 5.08 ± 1.54 under PE + Cd (75% reduction). Female adult fecundity followed a similar pattern: CK females laid 35.5 ± 0.15 eggs, whereas PE, Cd, and PE + Cd groups produced only 21.13 ± 0.41 (40% reduction), 20.98 ± 1.92 (41% reduction), and 19.06 ± 0.31 (46% reduction) eggs, respectively (all *p* < 0.001 compared to CK). These results demonstrate a combined inhibitory effect of PE and Cd exposure.

### 3.3. Age-Stage-Specific Survival Rate

**Figure 1:**
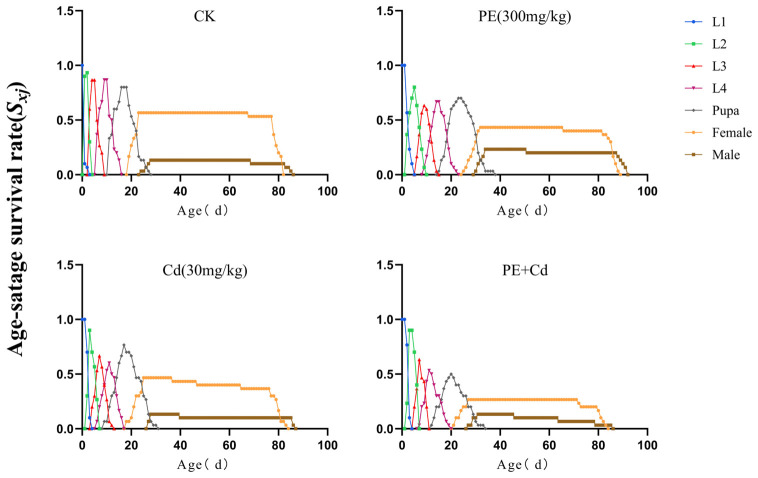
Age-stage-specific survival rate (*S_xj_*) of *Leptinotarsa decemlineata* on cadmium and/or microplastic-stressed potato plants.

The survival curve of the control group (CK) was generally relatively flat, exhibiting only slight declines during interstage transitions (Figure 1). By contrast, the PE + Cd combined application group displayed the lowest survival rates across all developmental stages. The first-instar larval survival rate in the combined application group was only 70% of that in the control group. The survival rate of L4 to the pupal stage was 25% lower than that of the control group, but 22% lower for the pupal stage to the adult stage. Additionally, adult longevity in the combined application group was shortened by 14 days relative to the control group.

### 3.4. Age-Specific Survivability and Age-Stage-Specific Fecundity

**Figure 2:**
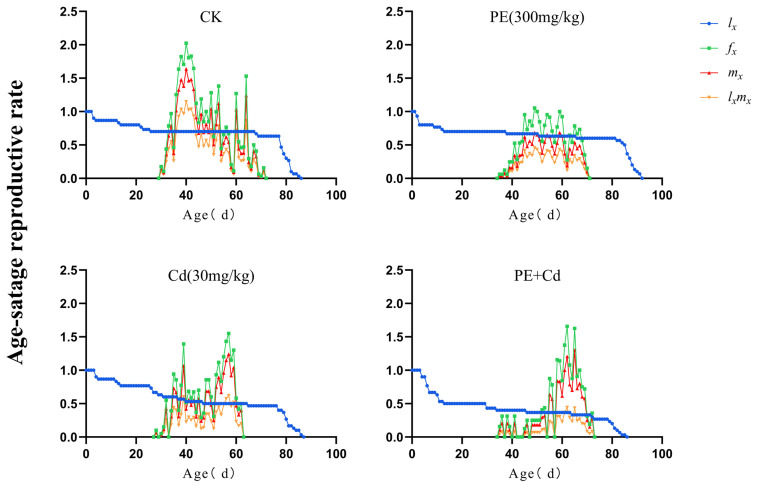
Age-specific survival rate (*l_x_*), female age-specific fecundity (*f_x_*), age-specific fecundity of the total population (*m_x_*), and age-specific maternity (*l_x_m_x_*) of *Leptinotarsa decemlineata* on cadmium and/or microplastic-stressed potato plants.

All treatment groups, especially the PE + Cd combined application group, showed delayed oviposition onset, lower fecundity peaks and shorter reproductive cycles. The combined treatment postponed the reproductive peak and markedly reduced fecundity, which agreed with the decline in female fecundity (Figure 2). The pollutants impaired reproductive performance via two mechanisms: delayed reproduction and decreased reproductive output per unit time.

### 3.5. Age-Stage-Specific Life Expectancy

**Figure 3:**
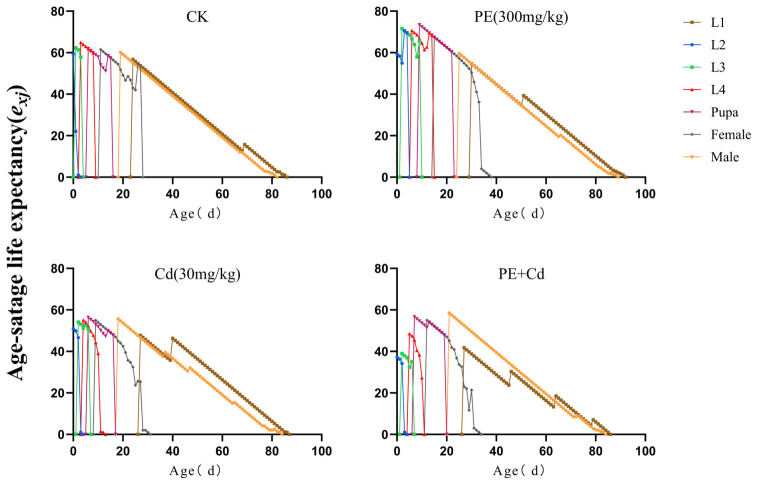
Age-stage life expectancy (*e_xj_*) of *Leptinotarsa decemlineata* on cadmium and/or microplastic-stressed potato plants. Note: L1–L4 represent the first, second, third, and fourth larval instars, respectively.

During larval stages, all treatment groups had consistently lower e_xj_ values than CK (Figure 3). For example, at age 10 d (L2–L3 stage), life expectancy in CK was approximately 38 remaining days, whereas PE + Cd reduced this to 24 days (a 37% reduction). This corresponds with the early survival decline in Figure 1, indicating that beetles in treatment groups experienced physiological stress beginning in the larval stage.

### 3.6. Age-Stage-Specific Reproductive Value

**Figure 4:**
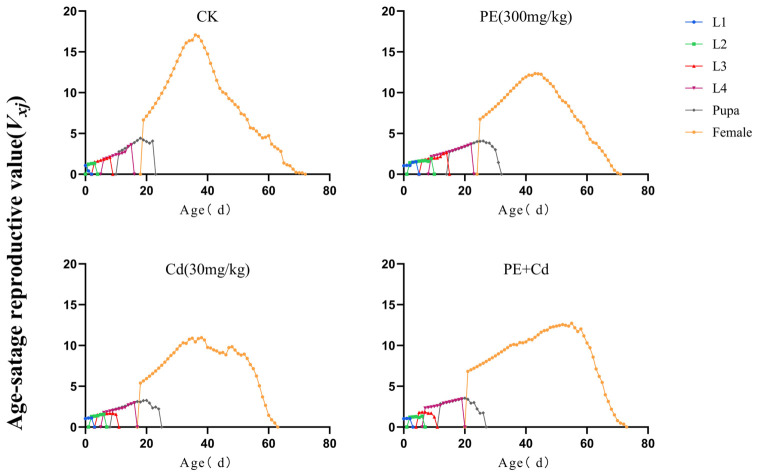
Age-stage reproductive value (*v_xj_*) of *Leptinotarsa decemlineata* on cadmium and/or microplastic-stressed potato plants. Note: L1–L4 represent the first, second, third, and fourth larval instars, respectively.

The curve for the control group (CK) increased rapidly and reached a pronounced peak (Figure 4). By contrast, the reproductive value curves of all treatment groups exhibited markedly lower peak values and were shifted to the right. This pattern indicates that individuals in the treatment groups had reduced total potential future contributions to population growth, and the peak period of this contribution was delayed.

## 4. Discussion

This study systematically elucidated the individual and combined toxic effects of polyethylene microplastics (PE) and cadmium (Cd) on the *Leptinotarsa decemlineata* using the two-sex life table. The primary findings revealed that exposure to either PE or Cd significantly delayed the beetle’s growth and development, reduced its survival and fecundity, and consequently suppressed its intrinsic potential for population increase. Notably, the combined treatment (PE + Cd) exhibited a combined inhibitory effect on most key parameters, rather than a simple additive effect.

The results align with existing reports on how environmental pollutants delay insect development and reduce reproductive capacity. For instance, Cd has been shown to disrupt energy metabolism and molting processes in insects by inducing oxidative stress, leading to developmental delays [26]. Microplastics, on the other hand, impair nutrient absorption through physical blockage of the gut and act as carriers that enhance the bioavailability of other toxicants [27]. In this study, PE treatment demonstrated a stronger effect than Cd in prolonging larval development (e.g., in the L2 stage, as shown in Table 1). This effect might be attributable to possible physical damage caused by microplastics to the chewing mouthparts or digestive tract, although we did not perform histological examinations to confirm this. Future studies using gut histology or ultrastructure analysis are needed to test this hypothesis.

The core finding of this research is highlighted by the combined effect of PE and Cd. The reduction in the intrinsic rate of increase(r) in the combined treatment (PE + Cd) group (59%) was substantially greater than the sum of the individual effects of PE (38%) and Cd (27%). Similarly, the net reproductive rate (*R*_0_) and female adult fecundity reached their lowest levels in this group. These results indicate that the combined application of PE and Cd exerts significant physiological stress on the *Leptinotarsa decemlineata*; such a shift may be attributed to a stress-induced reallocation of energy, whereby insects under adverse conditions invest more resources in maintenance and survival at the expense of reproduction. Consequently, this delay and reduction in reproductive output weaken the generational turnover rate and the population’s overall growth momentum.

Given that we did not directly measure Cd or PE accumulation in plant or insect tissues, the following mechanisms are speculative and presented as hypotheses to guide future research: It is plausible that PE microplastics adsorb Cd ions in the soil and that both are co-ingested by the beetle larvae [28]. Within the gut environment, the carrier effect of PE may facilitate the release and absorption of Cd [29,30]. Concurrently, the physical damage inflicted by PE on the intestinal structure likely compromises the insect’s detoxification and barrier functions. These combined actions could ultimately amplify the cellular toxicity of Cd (e.g., oxidative damage and enzyme inhibition) [31,32]. The sharp decline in early stage survival rates (Figure 1 and Figure 3) and the profound suppression of reproductive potential (Figure 2 and Figure 4) observed in the PE + Cd group collectively support this “carrier-amplification” hypothesis.

## 5. Conclusions

Our results notwithstanding, we briefly highlight the limitations of this study. Our work primarily confirms the combined toxicity of PE and Cd at the phenomenological and population levels. The precise in vivo toxicokinetic processes and underlying molecular mechanisms—such as their effects on the antioxidant system, ecdysone signaling pathways, and vitellogenin gene expression—remain unclear. Therefore, future research should focus on: (1) elucidating the interactive targets of PE and Cd at the cellular and molecular levels; (2) investigating the effect spectra of microplastics of different sizes and polymer types combined with various heavy metals; and (3) validating the general applicability of these laboratory findings within more complex, field-representative ecosystems.

It should be noted that our study used laboratory-reared insects fed on contaminated leaves only during the experimental generation. While this design minimizes background variability, it does not capture the effects of chronic, multigenerational exposure that may occur in polluted agricultural fields. Future studies should investigate transgenerational effects to better understand ecological consequences.

It should be acknowledged that this study was conducted under strictly controlled laboratory conditions. While such conditions allow for the establishment of clear causal relationships by minimizing environmental noise, they inevitably have limitations. Laboratory settings cannot fully replicate the heterogeneity of field soils, seasonal climatic fluctuations, complex microbial interactions, or the presence of natural enemies. Moreover, microplastic and cadmium contamination in real agricultural soils typically involves long-term, low-dose, combined exposure, whose ecological effects may differ from those observed in short-term, high-concentration laboratory experiments. Therefore, the findings of this study cannot be directly extrapolated to complex field ecosystems. Future research should prioritize semi-field and full-field validation experiments, complemented by long-term monitoring, to comprehensively assess the realistic impacts of combined microplastic and cadmium pollution on *Leptinotarsa decemlineata* population dynamics. Such efforts will provide a more robust ecological risk basis for pest early warning and integrated management in contaminated agricultural landscapes.

## Figures and Tables

**Table 1:** Development duration of the *Leptinotarsa decemlineata* on cadmium and/or microplastic-stressed potato plants.

Developmental Stage	CK	PE	Cd	PE + Cd
L1	1.04 ± 0.04 ^b^	2.96 ± 0.22 ^a^	2.67 ± 0.09 ^a^	2.74 ± 0.08 ^a^
L2	2.31 ± 0.09 ^d^	4.78 ± 0.09 ^a^	3.08 ± 0.14 ^c^	3.5 ± 0.11 ^b^
L3	3.81 ± 0.15 ^b^	4.43 ± 0.16 ^a^	3.33 ± 0.13 ^c^	3.31 ± 0.15 ^c^
L4	5.83 ± 0.16 ^b^	6.86 ± 0.14 ^a^	4.61 ± 0.17 ^b^	6.6 ± 0.31 ^a^
Pupa	9.67 ± 0.26 ^c^	11.55 ± 0.18 ^a^	10.67 ± 0.28 ^ab^	10.5 ± 0.32 ^b^
Adult	57.48 ± 1.05 ^a^	54.6 ± 2.3 ^a^	50.67 ± 3.92 ^a^	50.25 ± 3.8 ^a^
APOP	10.47 ± 0.26 ^a^	10.92 ± 0.29 ^a^	10.36 ± 0.32 ^a^	11.43 ± 0.48 ^a^
TPOP	32 ± 0.41 ^c^	38.92 ± 0.59 ^a^	32.07 ± 0.65 ^bc^	38 ± 0.79 ^ab^

Note: TPOP (total pre-oviposition period)—duration from egg hatch to first oviposition, APOP (adult pre-oviposition period)—duration from adult emergence to first oviposition. Significant difference was analyzed using a paired-sample paired bootstrap test of TWOSEX—MS Chart. Data are mean ± SE. Different letters in the same row indicate a significant difference at the *p* < 0.05 level by the paired bootstrap test.

**Table 2:** Population parameters of *Leptinotarsa decemlineata* on cadmium and/or microplastic-stressed potato plants.

Population Parameters	CK	PE	Cd	PE + Cd
Intrinsic rate of increase (*r*) (d^−1^)	0.066 ± 0.0039 ^a^	0.041 ± 0.0042 ^b^	0.049 ± 0.005 ^b^	0.027 ± 0.0058 ^c^
Finite rate of increase (*λ*) (d^−1^)	1.07 ± 0.0042 ^a^	1.042 ± 0.0044 ^b^	1.05 ± 0.0052 ^b^	1.028 ± 0.0059 ^c^
Net reproduction rate (*R*_0_)	20.12 ± 3.19 ^a^	9.16 ± 1.9133 ^b^	9.79 ± 2.095 ^b^	5.08 ± 1.54 ^b^
Gross reproduction rate (*GRR*)	28.78 ± 2.98 ^a^	14.26 ± 2.28 ^b^	18.6277 ± 2.34 ^b^	14.29 ± 2.46 ^b^
Fecundity (eggs·female^−1^·day^−1^)	35.5 ± 0.15 ^a^	21.13 ± 0.41 ^b^	20.98 ± 1.92 ^bc^	19.06 ± 0.311 ^c^

Note: “Fecundity” refers to the average number of eggs laid per day by each female insect during the egg-laying period. Significant difference was analyzed using a paired-sample paired bootstrap test of TWOSEX—MS Chart. Data are mean ± SE. Different letters in the same row indicate a significant difference at the *p* < 0.05 level by the paired bootstrap test.

## Data Availability

The original contributions presented in this study are included in the article. Further inquiries can be directed to the corresponding author.

## References

[1] Özsari P., Karsavuran Y. (2025). Feeding behavior of *Leptinotarsa decemlineata* (Say) (Coleoptera: Chrysomelidae) (*Leptinotarsa decemlineata*) and the amount of damage on potato plant. Appl. Ecol. Environ. Res..

[2] Shen H.F., Cao H.M., Ding G.H., Li J.T., Yang R.W., Liu Y. (2025). Occurrence patterns and control technologies of major diseases and pests of potato in Xinjiang. South China Agric..

[3] Liu X., Yang H., Niu F., Sun H., Li C. (2023). Impact of water stress on the demographic traits and population projection of *Leptinotarsa decemlineata*. Front. Physiol..

[4] Li F.P., Yang X.Y., Zhang Z.M., Jiang Y.C., Gong Y.F. (2024). Behaviour, ecological impacts of microplastics and cadmium on soil systems: A systematic review. Environ. Technol. Innov..

[5] Huang F.Y., Chen L., Yang X., Jeyakumar P., Wang Z., Sun S.Y., Qiu T.Y., Zeng Y., Chen J., Huang M. (2024). Unveiling the impacts of microplastics on cadmium transfer in the soil-plant-human system: A review. J. Hazard. Mater..

[6] Chen M.J., Khan A.R., Memon M.S., Iqbal B. (2025). Microplastics and nanoplastics across the food web: Challenges and mitigation strategies in securing human health. Process Saf. Environ. Prot..

[7] Wael H., Vanessa E.B., Mantoura N., Antonios D.E. (2025). Tiny pollutants, big consequences: Investigating the influence of nano- and microplastics on soil properties and plant health with mitigation strategies. Environ. Sci.-Process. Impacts.

[8] Liu S.M., Chen F.T., Wang C.H., Kong F.L., Jiang Z.X. (2025). Effects of polyethylene microplastics with different particle sizes on soil organic carbon characteristics and mineralization in agricultural soil. Soils.

[9] Zhang J.B., Hu Y., Niu F.S., Sun H.H., Li C. (2025). Adverse effects of polyethylene microplastics on the growth, development and predation of *Arma chinensis*. Chin. J. Appl. Entomol..

[10] Zhang J.B. (2025). Effects of Polyethylene Microplastics on the Development and Predation of *Leptinotarsa decemlineata* and Natural Enemy Stinkbug. Master’s Thesis.

[11] Zhao S.Q., Miao W.L., Sheng S., Pan X., Li P., Wu F.A. (2025). Effects of Cd exposure on physiological, biochemical and tissue damage of *Glyphodes pyloalis* Walker larva. Acta Sericologica Sin..

[12] He B., Zhang J., Hu Y., Zhang Y., Wang J., Li C. (2025). Age-stage, two-sex life table of *Leptinotarsa decemlineata* (Coleoptera: Chrysomelidae) experiencing cadmium stress. Insects.

[13] Song J., Sun Z., Saud S., Fahad S., Nawaz T. (2025). Exploring the deleterious effects of heavy metal cadmium on antioxidant defense and photosynthetic pathways in higher plants. Plant Stress.

[14] Liang B., Ye Q., Shi Z. (2024). Stable isotopic signature of cadmium in tracing the source, fate, and translocation of cadmium in soil: A review. J. Hazard. Mater..

[15] Khan M.M., Wang J., Gao Y., Wu D., Qiu B., Zhu Z. (2025). Impact of long-term cadmium exposure on insecticidal cross-resistance and biological traits of Brown planthopper *Nilaparvata lugens* (Hemiptera: Delphacidae). J. Hazard. Mater..

[16] Khan M.M., Fan Z.Y., Wang X.M., Qiu B.L. (2024). Distribution and accumulation of Cadmium in different trophic levels affecting *Serangium japonicum*, the predatory beetle of whitefly *Bemisia tabaci*, biologically, physiologically and genetically: An experimental study. J. Hazard. Mater..

[17] Emre I., Kayis T., Coskun M., Dursun O., Cogun H.Y. (2013). Changes in antioxidative enzyme activity, glycogen, lipid, protein, and malondialdehyde content in cadmium-treated *Galleria mellonella* larvae. Ann. Entomol. Soc. Am..

[18] Guo R.Y., Bo L.J., Li B., Jin W.Z., Li Y., Chai C., Wang Y.Q. (2025). Advances on combined pollution, migration characteristics and ecological effects of microplastics and cadmium in farmland soils. Acta Agric. Jiangxi.

[19] Zhang L.F., Zhao B.W., Zhu Z.Y., Zhang Y., Li Y.Q. (2025). Effects of polypropylene microplastics and cadmium on rhizosphere soil and microbial communities of maize seedlings and their mechanisms. Res. Environ. Sci..

[20] John A., Khan M.A., Mashlawi A.M., Kumar A., Rahayuningsih S., Wuryantini S., Endarto O., Gusti Agung Ayu Indrayani I., Suhara C., Rahayu F. (2025). Environmental contaminants and insects: Genetic strategies for ecosystem and agricultural sustainability. Sci. Total Environ..

[21] Luo Y.M., Zhou Q., Zhang H.B., Pan X.L., Tu C., Li L.Z., Yang J. (2018). Pay attention to research on microplastic pollution in soil for prevention of ecological and food chain risks. Bull. Chin. Acad. Sci..

[22] Ren S.Y., Wang K., Zhang J.R., Li J.J., Zhang H.Y., Qi R.M., Xu W., Yan C.R., Liu X.J., Zhang F.S. (2024). Potential sources and occurrence of macro-plastics and microplastics pollution in farmland soils: A typical case of China. Crit. Rev. Environ. Sci. Technol..

[23] (2009). Potato Beetle Outbreak Surveillance Protocols.

[24] Chi H., You M., Atlıhan R., Smith C.L., Kavousi A., Özgökçe M.S., Güncan A., Tuan S.J., Fu J.W., Xu Y.Y. (2020). Age-Stage, two-sex life table: An introduction to theory, data analysis, and application. Entomol. Gen..

[25] Chi H. (1988). Life-table analysis incorporating both sexes and variable development rates among individuals. Environ. Entomol..

[26] Sun J., Wu J., Liu F., Wei Q., Kang W., Zhao M., Xu S., Han B. (2025). Insights into physiological, reproductive, and transgenerational toxicity induced by larval cadmium exposure in honeybee (*Apis mellifera*) queens. Environ. Sci. Technol..

[27] Song X.J. (2025). Driving Mechanisms of Phosphorus Migration and Transformation Mediated by Microplastics in Freshwater Ecosystems. Master’s Thesis.

[28] Ritchie M.W., Provencher J.F., Allison J.E., Muzzatti M.J., MacMillan H.A. (2024). The digestive system of a cricket pulverizes polyethylene microplastics down to the nanoplastic scale. Environ. Pollut..

[29] Turna Demir F., Akkoyunlu G., Demir E. (2022). Interactions of Ingested Polystyrene Microplastics with Heavy Metals (Cadmium or Silver) as Environmental Pollutants: A Comprehensive In Vivo Study Using *Drosophila melanogaster*. Biology.

[30] Zhao T.W., Bai J., Wang J., Xiao Y., Zhao Y.H., Wu X.G., Zhang W.H. (2025). Response of Black Soldier Fly larvae development and Gut Microbial composition to Microplastics. Environ. Sci. Technol..

[31] Zhou Y., Yang Y., Liu G., He G., Liu W. (2020). Adsorption mechanism of cadmium on microplastics and their desorption behavior in sediment and gut environments: The roles of water pH, lead ions, natural organic matter and phenanthrene. Water Res..

[32] Soldano S., Bonanomi M., Aramini T., Moyano A., Garbelli A., Croce A.C., Weththimuni M.L., Vaghi P., Puggioli A., Gomulski L.M. (2025). Tracking micro- and nanoplastics in *Aedes albopictus*: From ingestion to metabolic disruption. Sci. Total Environ..

